# Clinical relevance of transcranial Doppler in a cardiac surgery setting: embolic load predicts difficult separation from cardiopulmonary bypass

**DOI:** 10.1186/s13019-024-02591-4

**Published:** 2024-02-13

**Authors:** Stéphanie Jarry, Etienne J. Couture, William Beaubien-Souligny, Armindo Fernandes, Annik Fortier, Walid Ben-Ali, Georges Desjardins, Karel Huard, Tanya Mailhot, André Y. Denault

**Affiliations:** 1 Department of Anesthesiology, Montreal Heart Institute, Université de Montréal, 5000 Belanger Street, Montreal, QC H1T 1C8 Canada; 2 Department of Anesthesiology and Department of Medicine, Division of Intensive Care Medicine, Institut Universitaire de Cardiologie et de Pneumologie de Québec, Quebec, QC Canada; 3 Department of Nephrology, Centre Hospitalier de l’Université de Montréal, Montreal, QC Canada; 4 Perfusion Service, Montreal Heart Institute, Université de Montréal, Montreal, QC Canada; 5 Montreal Health Innovations Coordinating Center, Montreal Heart Institute, Montreal, QC Canada; 6 Department of Surgery and Department of Cardiology, Montreal Heart Institute, Montreal, QC Canada; 7 Université de Montréal, Montreal, QC Canada; 8 Research Center, Montreal Heart Institute, and Faculty of Nursing, Université de Montréal, Montreal, QC Canada

**Keywords:** Transcranial Doppler, Cardiac surgery, Cardiopulmonary bypass, High intensity transient signal, Cerebral microemboli

## Abstract

**Background:**

During cardiac surgery, transcranial Doppler (TCD) represents a non-invasive modality that allows measurement of red blood cell flow velocities in the cerebral arteries. TCD can also be used to detect and monitor embolic material in the cerebral circulation. Detection of microemboli is reported as a high intensity transient signal (HITS). The importance of cerebral microemboli during cardiac surgery has been linked to the increased incidence of postoperative renal failure, right ventricular dysfunction, and hemodynamic instability. The objective of this study is to determine whether the embolic load is associated with hemodynamic instability during cardiopulmonary bypass (CPB) separation and postoperative complications.

**Methods:**

A retrospective single-centre cohort study of 354 patients undergoing cardiac surgery between December 2015 and March 2020 was conducted. Patients were divided in tertiles, where 117 patients had a low quantity of embolic material (LEM), 119 patients have a medium quantity of microemboli (MEM) and 118 patients who have a high quantity of embolic material (HEM). The primary endpoint was a difficult CPB separation. Multivariate logistic regression was used to determine the potential association between a difficult CPB separation and the number of embolic materials.

**Results:**

Patients who had a difficult CPB separation had more HITS compared to patients who had a successful CPB separation (p < 0.001). In the multivariate analysis, patients with MEM decreased their odds of having a difficult CPB weaning compared to patients in the HEM group (OR = 0.253, CI 0.111–0.593; p = 0.001). In the postoperative period patients in the HEM group have a higher Time of Persistent Organ Dysfunction (TPOD), a longer stay in the ICU, a longer duration under vasopressor drugs and a higher mortality rate compared to those in the MEM and LEM groups.

**Conclusion:**

The result of this study suggests that a high quantity of cerebral embolic material increases the odds of having a difficult CPB separation. Also, it seems to be associated to more complex surgery, a longer CPB time, a higher TPOD and a longer stay in the ICU. Six out of eight patients who died in this cohort were in the HEM group.

**Supplementary Information:**

The online version contains supplementary material available at 10.1186/s13019-024-02591-4.

## Introduction

During cardiac surgery, transcranial Doppler (TCD) represents a non-invasive modality that allows measurement of cerebral artery blood flow velocities (CBFV), cerebral autoregulation and the monitoring of micro embolic load, reported as a high-intensity transient signal (HITS) [1]. The presence of HITS on TCD has been reported to result from both gaseous and solid emboli [2].

Gas embolism is an important complication in cardiac surgery that can lead to increased risk of stroke, myocardial infarction and death [3, 4]. Emboli of a solid nature can occur during cardiac surgery. It is observed during aortic manipulation mostly in patients with documented aortic atheroma [5].

The occurrence of cerebral microemboli during cardiac surgery, until now, has been associated in limited reports with increased incidence of postoperative renal failure [6], right ventricular dysfunction [7, 8] and neurological dysfunction [9]. Microemboli affecting the cerebral circulation are believed to mediate postoperative cognitive dysfunction and stroke, in a proportion of cardiac surgery patient [10–12]. The relationship between the amount of HITS and non-neurological postoperative complications other that acute kidney injury has not been explored.

The objective of the study is to determine whether the embolic load in cardiac surgery is associated with hemodynamic instability upon weaning from cardiopulmonary bypass (CPB) separation and post-operative complications. We hypothesized that greater embolic load is associated with more difficult CPB separation and with prolonged vasoactive support postoperatively. [13–16]

## Methods

### Setting and study population

After approval by the local research and ethics committee (#2021–2810), a retrospective single-centre cohort study of 354 patients undergoing cardiac surgery in which routine TCD was used between February 2015 and June 2021 was conducted. For this study, two independent investigators screened a hemodynamic/echocardiographic database [17, 18] for consecutive patients undergoing cardiac surgery with TCD monitoring. Patients undergoing non-cardiac procedures, percutaneous procedures, or left ventricular assist device insertion as the primary procedure were excluded from the study.

At our centre, anesthetic induction included propofol and fentanyl. Tracheal intubation is facilitated with rocuronium and maintenance of anesthesia is performed with a combination of isoflurane or sevoflurane, fentanyl and propofol. During usual care, the use of vasoactive agents during CPB weaning is systematized based on an algorithm for intraoperative vasoactive management [13]. Intraoperative patients’ management was the same between the 3 groups. Other monitoring techniques include the use of transesophageal echocardiography, near-infrared spectroscopy (NIRS) and processed electroencephalography (pEEG) [17].

### Data collection

Demographic and baseline clinical data were collected from patient medical records. Data collected include age, sex, weight, cardiovascular risk factors as well as the type of surgery performed, the New York Heart Association (NYHA) functional classification grade and the European System for Cardiac Operative Risk Evaluation II (EuroSCORE II). Intraoperative data were extracted from the electronic patient record Compurecord Peri-Operative System (Version G.01 2015; Philips Healthcare, The Netherlands) and included fluid balance, blood loss, CPB separation classification, duration of CPB and aortic cross-clamping, brain oximetry, patient hemodynamic data before CPB and HITS. The type of oxygenator used for each patient undergoing cardiac surgery on CPB was identified. Postoperative data were collected from patient’s medical record and included the duration of the intensive care unit (ICU) and hospital stay, duration of mechanical ventilation, time of persistent organ dysfunction (TPOD) and duration of vasopressor requirements. Detailed definitions can be found in Additional File 1: Table S1.

The primary endpoint was difficult CPB separation defined by the use of at least two different classes of vasoactive agents (inotropes or inhaled vasodilators and vasopressors), a return on CPB, or the use of a mechanical circulatory support, such as an intra-aortic balloon pump, a left ventricular assist device or an extracorporeal membrane oxygenation. A successful separation from CPB was defined as the use of ≤ 1 vasopressor or ≤ 1 inotrope to achieve CPB separation (Additional File 2: Fig. S1) [13].

### Transcranial Doppler

In this study, the Digital TCD system (Model PMD150, Spencer Technologies, Seattle, WA, USA) was used (Additional File 2: Fig. S2). To facilitate the identification of the temporal acoustic window two-dimensional (2D) bedside brain ultrasound is used in the operating room [19]. The best signal on the right and on the left of the brain were selected for monitoring. The TCD signals are obtained with a 2 MHz probe frequency, using the 33-gate M-mode colour display. By using pulsed-wave ultrasound, TCD allowed the detection of HITS. On the TCD, a HITS is characterized by is unidirectional random occurrence with less than 300 ms duration and amplitude of more than 3 dB above the background intensity and causing a characteristic spike on the Doppler spectrum (Additional File 2: Fig. S2) [20]. The number of HITS, both on the right and the left side, is automatically calculated and entered in the database by the attending anesthesiologist. The maximum amount of HITS per patients was computed until the chest closure.

### Statistical analysis

In order to present the data, patients were divided in tertiles, where 117 patients had a low quantity of embolic material (LEM), 119 patients have a medium quantity of embolic material (MEM) and 118 patients who have a high quantity of embolic material (HEM). Low, medium, and high HITS groups correspond to patients who had below 133 HITS, between 133 and 413 HITS and more than 413 HITS, respectively. Descriptive statistics of demographics, hemodynamics, intraoperative and postoperative period were presented according to the normality of distribution of each variable. The distribution of the continuous variables was assessed using plots, normality tests and skewness and kurtosis index. Continuous variables were summarized using mean and standard deviation, or median and Q1–Q3, according to the distribution of variables. Categorical variables were described using frequencies and percentages. Group comparisons were made using one-way analysis of variance (ANOVA), Kruskal–Wallis tests or chi-square tests where appropriate. The global test comparing the three groups was produced first and if statistically significant, 2-by-2 comparisons between groups were done. The potential association between the number of HITS (exposure) and a difficult CPB separation (outcome) was analyzed using simple and multiple logistic regression models. Of the 354 patients in this study, 41 underwent a beating heart surgery and were not included in these models. Because of technical limitations, the amount of HITS could not be monitored in 57 patients. The regression model was therefore performed on 306 patients after excluding 41 patients who underwent off pump procedures and 7 patients with missing data (Additional File 2: Fig. S3). Because the assumption of linearity between the continuous variable HITS and the logit of difficult CPB separation was violated, HITS were then categorized into three groups according to the tertiles and analyzed and presented following this new format.

Age, procedure type, CPB duration, EuroSCORE II, central venous pressure (CVP) before CPB, and left ventricular ejection fraction (LVEF) before the surgery were included in the multiple regression models as adjustment terms. Odds ratios (OR) are presented along with their 95% confidence interval (95% CI) and p-value. A two-tailed p value < 0.05 was considered statistically significant. There was no correction or adjustment for multiple testing. No imputation was done as there were few missing data. All statistical analyses were performed using SPSS for Mac version 25 (IBM Corp, Armonk, NY) and SAS 9.4 (SAS Institute, Cary, NC).

## Results

**Fig. 1:**
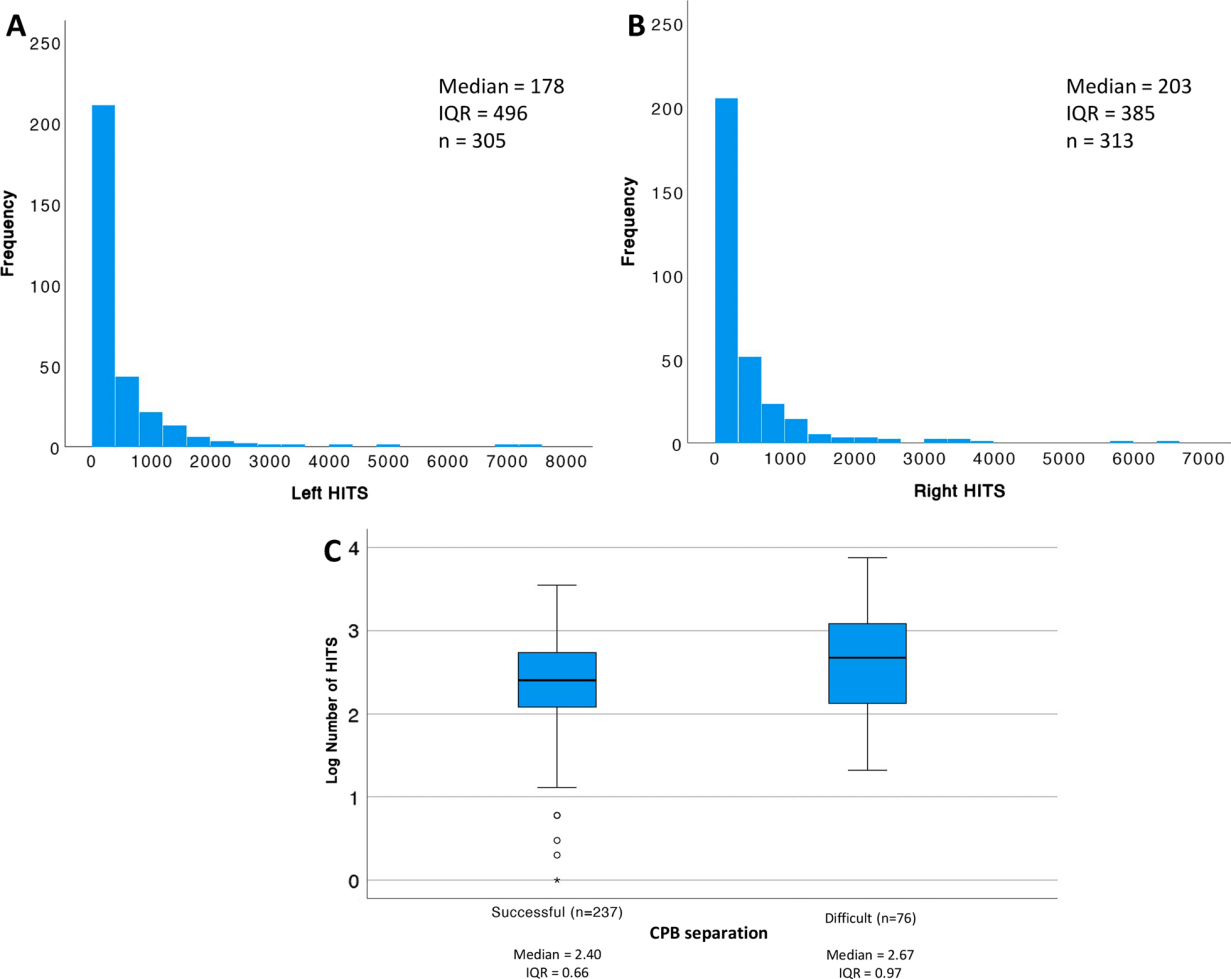
HITS distribution. **A** Left side HITS distribution. **B** Right side HITS distribution. **C** HITS distribution according to CPB separation classification, after log transforming the number of HITS. The log transformation allowed a better visual comparison. The horizontal line is the median value, the box represents the inter-quartile range, and the bars represent the upper and lower 25th percentiles, excluding outliers. Significant differences to control is indicated by an asterisk (P < 0.001). *CPB* cardiopulmonary bypass, *HITS* high-intensity transient signal, *IQR* interquartile range

**Fig. 2:**
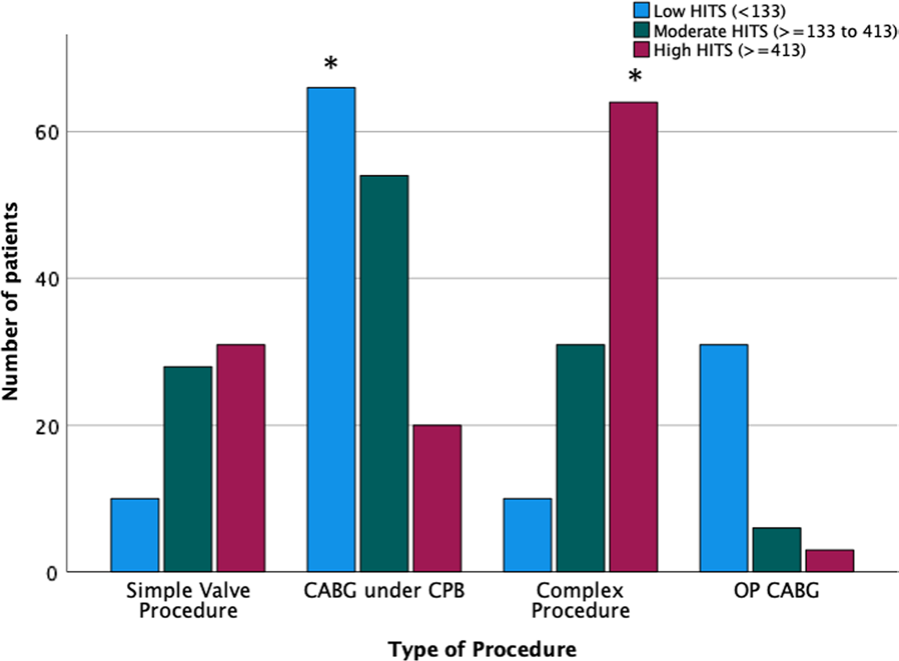
HITS distribution according to the type of procedure and stratify by HITS category. Significant differences to control are indicated by asterisks (P < 0.001). *CABG* coronary artery bypass graft, *CPB* cardiopulmonary bypass, *HITS* high-intensity transient signal, *OP* off pump

A total of 354 patients were included in the general analysis (Additional File 2: Fig. S3). The median number of HITS per patient was 242 [96.5–568.2]. In the cohort, 90 patients (25%) had a unilateral signal while 264 patients had a bilateral signal. The distribution of HITS on the left and right sides are shown in Fig. 1A, B. Demographic data, medical conditions and perioperative data of the population are summarized in Table 1. There was no difference between the three groups in terms of sex, body mass index (BMI), EuroSCORE II, New York Heart Association class III-IV, urgent procedure and comorbidities such as pulmonary hypertension, acute kidney injury and diabetes. On the other hand, patients in the LEM group are older (mean ± SD, LEM: 67.14 ± 10.36; MEM: 63.64 ± 12.50; HEM: 64.42 ± 11.36, p = 0.05) and have more hypertension (LEM: 77.8%; MEM: 59.7%; HEM: 61%, p = 0.005). Patients in the HEM group have more complex surgery (LEM: 9%; MEM: 26%; HEM: 54%, p < 0.001) and more single valve replacement procedure (LEM: 8.5%; MEM: 23.5%; HEM:26.3%, p < 0.001). Patients undergoing coronary artery bypass graft (CABG) procedures with (LEM: 56.4%; MEM: 45.4%; HEM: 16.9%, p < 0.001), or without CPB (LEM: 26.5%; MEM: 5%; HEM: 3.4%, p < 0.001) had fewer microemboli during the surgery (Fig. 2).

**Table 1:** Demographic and preoperative characteristics of the study population

	LEM (n = 117) (Below 133)	MEM (n = 119) (≥ 133 to 413)	HEM (n = 118) (≥ 413)	*P* value
Age (years)	67.14 ± 10.36	63.64 ± 12.50	64.42 ± 11.36	0.050^*^
Male	102(87.5)	95(79.8)	94(79.7)	0.228
BMI	28.76 ± 4.49	28.82 ± 5.58	28.52 ± 5.08	0.891
EuroSCORE II	1.47[0.9–3.1]	1.50[0.9–2.8]	1.60[1.1–4.55]	0.102
New York Heart Association III–IV	15(11.2)	12(10.1)	8(6.8)	0.299
Comorbidities				
Pulmonary hypertension^a^	22(18.8)	22(18.5)	32(27.1)	0.187
Acute kidney injury^a^	13(11.1)	8(6.7)	12(10.2)	0.474
Diabetes mellitus	40(34.2)	33(27.7)	33(28)	0.472
Arterial hypertension	91(77.8)	71(59.7)	72(61.0)	0.005^†,‡^
Chronic obstructive pulmonary disease	7(6)	6(5)	7(5.9)	0.940
Heart failure with reduced ejection fraction^a^	30(25.6)	28(23.9)	26(22.2)	0.829
Peripheral vascular disease	30(25.6)	25(21.0)	20(16.9)	0.264
Procedures				
Urgent surgery^a^	26(22.2)	19(16.0)	19(16.1)	0.363
Coronary artery bypass graft with CPB	66(56.4)	54(45.4)	20(16.9)	< 0.001^§^
Coronary artery bypass graft without CPB	31(26.5)	6(5.0)	4(3.4)	< 0.001^§^
Simple valve	10(8.5)	28(23.5)	31(26.3)	0.001^§^
Complex surgery^a^	10(8.5)	31(26.1)	64(54.2)	< 0.001^§^
Intraoperative data				
Cardiopulmonary bypass duration (min)^b^	67[51–94.75]	74[53.50–99.50]	98[72.75–136]	< 0.001^†,‡^
Cross-clamping time (min)^b^	44[33.5–67]	51[37.5–77.5]	74[56.25–106.5]	< 0.001^†,‡^
CPB Fusion membrane^b^	24(20.5)	40(33.6)	69(58.5)	< 0.001^§^
Intraoperative fluid balance (mL)	770[232.5–1275]	745[350–1255]	632.5[− 18.25–1290]	0.350

^a^See Additional File 1: Table S1

^b^Only 306 patients undergoing CPB were included for theses analysis. Low (n = 86), moderate (n = 113) and high HITS (n = 114) group correspond to patients who had below 161 HITS, > 173 and 473 HITS and > 473 HITS, respectively

^*^Difference between the LEM and MEM group

^†^Difference between the LEM and HEM group

^‡^Difference between the HEM and MEM group

^§^Difference between the 3 groups

*BMI* body mass index, *EuroSCORE II* European System for Cardiac Operative Risk Evaluation, *HEM* high quantity of embolic material, *HITS* high-intensity transient signal, *LEM* low quantity of embolic material, *MEM* medium quantity of embolic material

In terms of intraoperative data (Table 1), patients in the HEM group seem to experience longer CPB duration (median [IQR], HEM: 98 min [72.75–136]; MEM: 74 min [53.50–99.50]; LEM: 67 min [51–94.75], p < 0.001). The number of HITS in relation to the type of membrane oxygenators is shown in Additional File 2: Figs. S4 and S5. More HITS were observed in the Fusion type membrane oxygenator (Affinity Fusion, Medtronic, Minneapolis, USA) during the procedure (HEM: 58.5%; MEM: 33.6%; LEM: 20.5%, p < 0.001) (Table 1). However, no difference was observed between difficult CPB separation and the type of oxygenator (Additional File 1: Table S2).

In terms of intraoperative complications (Table 2), difficult CPB separation was observed in 35,1% of patients in the HEM group compared to 15.9% and 20.9% of patients in the MEM and LEM group respectively (p = 0.002). Patients who had a difficult CPB separation had more HITS compared to patients who had a successful CPB separation (p < 0.001) (Fig. 1C). Patients in the HEM group seem to experience more intraoperative bleeding (median [IQR], LEM: 350 mL [220–467.5]; MEM: 375 mL [215–500]; HEM: 400 mL [300–600]; p = 0.006). There was no difference in terms of cerebral desaturation during the procedure between the 3 groups. No difference in terms of hemodynamic variables were observed except higher cardiac output in the HEM group before CPB (mean ± SD, HEM: 4.08 ± 1.29; MEM: 3.86 ± 1.11; LEM: 3.60 ± 1.03, p = 0.013) (Additional File 1: Table S3).

**Table 2:** Peri-operative outcomes

	LEM (n = 86) (Below 133)	MEM (n = 113) (≥133–413)	HEM (n = 114) (≥ 413	*P* value
CPB Separation^a^				
Successful weaning	68(79.1)	95(84.1)	74(64.9)	0.002^*^
Difficult weaning	18(20.9)	18(15.9)	40(35.1)	
Intraoperative bleeding (mL)	350[220–467.5]	375[215–500]	400[300–600]	0.006^†,‡^
Brain desaturation in the operating room (%)^b^	37(32.2)	44(37.9)	46(40.4)	0.419
Defibrillation	24(20.5)	35(29.4)	38(32.2)	0.111

^a^Only 306 patients undergoing CPB were included for theses analysis. Low (n = 86), moderate (n = 113) and high HITS (n = 114) group correspond to patients who had below 161 HITS, >161 and 473 HITS and >473 HITS, respectively

^b^See Additional File 1: Table S1

^*^Difference between the 3 groups

^†^Difference between the Low HITS and High HITS group

^‡^Difference between the High and Moderate HITS group

*CPB* cardiopulmonary bypass, *HEM* high quantity of embolic material, *LEM* low quantity of embolic material, *MEM* medium quantity of embolic material

In the postoperative period, patients in the HEM group have significantly higher TPOD (median [IQR], HEM: 16[3–40.5]; MEM: 6[2–23]; LEM: 9[2–22.5], p < 0.001), a longer duration of vasopressor requirements (median [IQR], HEM: 16 h[3–40.5]; MEM: 6 h[2–23]; LEM: 9 h[2–22.5], p = 0.013), a longer ICU stay (median [IQR], HEM: 2 days [1–4]; MEM: 2 days [1–3]; LEM: 1 day [1–3], p = 0.022) and higher mortality rate (HEM:5.1%; MEM: 1.7%; LEM:0%, p = 0.028) compared to those in the MEM and LEM groups (Table 3). Among the 8 patients who died, 6 were in the HEM group. Characteristics of deceased patients are presented in Additional File 1: Table S4. However, there was no difference in terms of hospital length of stay (Table 3).

**Table 3:** Postoperative outcomes

	LEM (n = 117) (Below 133)	MEM (n = 119) (≥ 133 and 413)	HEM (n = 118) ≥ 413)	*P* value
Duration of mechanical ventilation (h)	3[2–5]	3[2–4]	3[2–5.25]	0.483
TPOD (h)^a^	9[4-24]	7[3–27]	21.5[6.75–50.25]	< 0.001*^†^
Acute kidney injury (%)^a^	41(35)	36(30.3)	49(41.5)	0.191
Vasopressor time (h)^a^	9[2–22.5]	6[2–23]	16[3–40.5]	0.013 *^†^
Length of stay in the ICU (days)	1[1–3]	2[1–3]	2[1–4]	0.022 ^†^
Length of hospital stay (days)	5[4–7]	5[5–7]	6[5–8]	0.157
Death	0(0)	2(1.7)	6(5.1)	0.028 ^†^

^a^See Additional File 1: Table S1

^*^Difference between the LEM and HEM group

^†^Difference between the High and Moderate HITS group

*CPB* cardiopulmonary bypass, *HEM* high quantity of embolic material, *HITS* high-intensity transient signal, *ICU* intensive care unit, *LEM* low quantity of embolic material, *MEM* high quantity of embolic material, *TPOD* Time with Persistent Organ Dysfunction

In the multivariate analysis, patients with MEM decreased their odds of having a difficult CPB weaning compared to patients in the HEM group (OR = 0.257, CI 0.111–0.593; p = 0.001) (Table 4). However, no association was observed between patients with LEM and HEM (OR = 0.705, CI 0.307–1.621; p = 0.410).

**Table 4:** Multiple analysis of risk factors of difficult CPB separation and higher HITS

Multivariable analysis of difficult CPB separation			
Risk factors	OR^a^	95% CI	P value
Lower HITS category^b^	0.687	0.299–1.576	0.376
Medium HITS category^b^	0.253	0.109–0.584	0.001
Simple valve replacement^c^	0.977	0.404–2.360	0.959
Coronary artery bypass graft with CPB^c^	1.157	0.485–2.765	0.742
Cardiopulmonary bypass duration (min)	1.008	1.002–1.014	0.007
EuroSCORE II	1.179	1.083–1.284	< 0.001
Central venous pressure before CPB (mmHg)	1.082	1.005–1.164	0.037
Left ventricular ejection fraction superior to 50%	0.451	0.221–0.922	0.029
Age (year)	0.977	0.952–1.003	0.085

^a^The odds ratio is calculated for a difference of one additional unit for continuous variables

^b^The reference category is High HITS category

^c^The reference is the complex surgery

*CI* confidence interval, *CPB* cardiopulmonary bypass, *EuroSCORE II* European System for Cardiac Operative Risk Evaluation, *HITS* high-intensity transient signal, *OR* odds ratio

## Discussion

This study suggests that patients with a high quantity of embolic material are more at risk of a difficult CPB separation when adjusted for other parameters. In fact, patients with high versus medium embolic load increased their odds of having a difficult CPB weaning. In addition, elevated embolic load seems to be associated with longer duration of vasoactive support in the ICU, excessive bleeding, prolonged organ dysfunction and higher mortality. This association could be explained by the local cardiac and systemic effects of both gaseous and solid emboli which can lead to local ischemia but also to inflammatory responses, complement and coagulation activation [21]. The difficult separation from CPB is most likely the result of embolization into the right coronary artery [7, 8] leading to right ventricular dysfunction [7] which if persists is associated with significant post-operative complications and increased mortality [13–16]. As mentioned previously, patients in the HEM group have more complex surgery. This could explain why patients in the HEM group had longer duration of vasoactive support in the ICU and a longer stay in the ICU.

In a cardiac surgery setting, the presence of emboli can occur before and during CPB. Before CPB, patients with an intracardiac shunt are more likely to present paradoxical embolism which is associated with stroke and systemic embolization [22, 23]. Emboli can also occur during CPB. It is well documented that both the use of CPB and aortic cannulation and manipulation produce a greater number of cerebral microemboli [24, 25]. Several studies have shown that off-pump CPB is associated with a reduction in the number of HITS which is consistent with our findings [26–29]. This may partially explain the observed reports of fewer transfusion requirements, shorter intubation time, reduced stroke [30] and ICU length of stay in off-CPB surgery. A small prospective study of 56 patients reported that patient who underwent cardiac surgery with a CPB duration more than 91 min increases by 6% the odds of acquiring new microemboli for each additional minute under CPB [31].

During CPB, the presence of emboli have been attributed to different reasons; perfusionists interventions [32], the use of negative pressure on the CPB venous line [33–35] and the CPB blood flow rate to name a few. The literature indicates that air in the CPB venous line is directly associated with arterial emboli. The presence of venous air is explained by different reasons ranging from residual air in the venous cannula [36], use of left ventricular vent [37], to air entrainment from the venous line caused by loss of the right atrial seal around the venous cannula following a surgical manipulation [32, 38]. Before or after aortic cross-clamp positioning, paradoxical embolism of venous air across atrial or ventricular septal defects can also occur.

We observed that a higher proportion of patients in the HEM group had an arterial CPB Medtronic fusion oxygenator compared to MEM and LEM group. Depending on the size of the gaseous microemboli, an in vitro study found that Medtronic Fusion oxygenator seems to be less effective compared to the Inspire oxygenator [39]. This might be explained by the difference in the oxygenator’s design. Despite this observation unreported so far, there were no differences in terms of separation from CPB between all the oxygenators.

It had been reported that a greater amount of large gas emboli is observed typically during valve procedures after CPB following release of the aortic cross-clamping compared to CABG surgery [31]. In this study, patient who underwent valve procedures had longer CPB time. We observed similar finding in our cohort regarding the number of HITS and the type of procedure. Open heart cardiac surgery has a higher risk of incomplete deairing of the cardiac cavities, explaining the increased number of HITS as described in the literature [33, 40].

As a complication of intraoperative emboli, we observed that patients in the HEM group seem to experience more difficult separation from CPB, intraoperative bleeding, longer ICU stay, longer duration of organ dysfunction and more deaths. A retrospective review of 286 patients found that increased number of HITS during CABG procedure was associated with postoperative renal failure [6]. However, we did not observe any significant association with acute kidney injury despite more 6 out of 8 deaths in the HEM group. This present study supports previous observations relating the amount of emboli with renal dysfunction which could result from direct emboli to the kidneys as previously observed using simultaneous renal ultrasound and TCD [41] or as a result of right heart failure from coronary embolization [7, 8, 42] leading to renal venous congestion and failure [43, 44]. This study represents so far the largest cohort in which high amount of HITS during cardiac surgery is associated with difficult separation from CPB and increased risk of postoperative complications. However, patients in the HEM group underwent more complex and longer surgery. The extent through which the high number of HITS observed in those patients is an independent predictor of TPOD remain to be explored.

During cardiac surgery, several techniques can be used to limit the amount of HITS in the operating room. Epiaortic scanning prior to cannulation has been recommended in order to identify severe atherosclerosis prior to cannulation [45]. An experienced operator can evaluate the entire ascending aorta thoroughly in less than five minutes [45]. Epiaortic scanning is mostly useful in detecting soft-lipid rich elements which are undetectable using finger palpation particularly if they are on the opposite or sidewall of the aorta [46]. Other indications for epiaortic scanning include age (> 60 years), calcified aortic knobs on chest radiograph or palpable calcifications in the ascending aorta, severe peripheral vascular disease and previous history of transient ischemic attack or cerebrovascular accident. Several precautions regarding surgical techniques can be used to limit the amount of HITS, such as minimizing the volume of air in the venous cannula after cannulation of the right atrium [36]. Following open heart surgery, Valsalva maneuver and de-airing are used to flush out residual air from the lungs and minimizing air from entering the pulmonary veins [47]. Also, the use of carbon dioxide insufflation into the pericardial cavity during cardiac surgery may reduce gaseous microemboli to the brain [48]. In our centre, the process of deairing is initiated by reducing venous drainage to a CVP of at least 10 mmHg. At this point pulmonary insufflation to achieve an airway pressure of 30 cmH2O, aortic root venting is initiated, CPB flow is reduced, and aortic cross-clamp is removed. Aortic root venting is continued during the period of full CPB support, and the duration of venting is decided by transesophageal echocardiographic (TEE) monitoring. The aortic root venting is stopped once CPB is weaned, unless there are still signs of air on TEE. At this point venting will be continued while the CPB support is null. Cerebral embolic protection devices, such as bubble traps are still under study, but seem to be interesting alternatives to limit the number of emboli during the procedure [49, 50].

This study has several limitations and confounders regarding the study design. It was a retrospective single-institution study where the routine use of TCD is still not well established in the practice of all anesthesiologists. Considered a basic skill in brain ultrasonography, identification of the middle cerebral artery (MCA) requires some practice [51]. One of the main limitations of TCD use is the ability to obtain an acoustic window which may be impossible in 5–20% of patients as a result of the bone thickness of the temporal bone [19, 52]. The use of hand-held ultrasound significantly improved our ability to identify and exclude those patients. Therefore we can select those in which a unilateral or bilateral acoustic window allows TCD monitoring with a 95% success rate [19]. Fully autonomous robotic TCD systems are currently available and may facilitate routine use of this modality. Our median number of HITS was slightly higher than Rodrigez et al. but they only included CABG patients [53] which represents 56.4% of our population. As a post-operative outcome, we have not included the vasoactive and inotropic score in our analyses. This data could have been relevant to quantify the degree of hemodynamic support for patient arrival in the ICU. As mentioned previously, patients in the HEM group have more complex surgery. This could explain why patients in the HEM group had longer duration of vasoactive support in the ICU and a longer stay in the ICU. At the authors' hospital, only one TCD is available, limiting patient monitoring. Also, we included all patients with TCD data available during a specific period. Management and clinical practice may have changed between February 2015 and June 2021. In our study, we did not include at what specific time the embolic load occurred. It has been reported that 2 periods associated with the highest number of HITS are the period from aortic cross-clamping to immediately before declamping and the period from the release of the aortic side clamp until the end of the CPB [53]. At this point, the current technology does not allow to distinguish between solid and gaseous embolic material or size distribution. In addition, we monitored non-selectively one of the available cerebral arteries which are from the anterior cerebral circulation such as the MCA. The emboli originating from the vertebral artery territory could be missed with MCA monitoring. The patients’ cognitive function in the preoperative and postoperative period was not addressed in this study. The impact of microemboli on postoperative cognitive decline and delirium, in the context of cardiac surgery remain controversial [31, 54]. We assume that patients in the LEM group had no advantage in their odds of having a difficult CPB weaning compared to patients in the HEM group because the embolic load in terms of risk factors may be too low to explain difficult CPB weaning. There may be other more important factors that could explain it. Finally, there are other roles for TCD such as detection of the adequacy of autoregulation, detection of cerebral venous congestion [55, 56], arterial malperfusion during aortic surgery [57] or endarterectomy [58] and its role as part of multimodal brain monitoring [59, 60] which makes TCD a non-invasive promising routine monitoring strategy in cardiac surgery.

## Conclusion

In cardiac surgery, a high quantity of cerebral embolic material appears to increase the odds of having a difficult CPB separation. Elevated number of cerebral emboli is present in patients with more complex surgery and a longer CPB time. Elevated number of HITS seems to be associated with longer duration of post-operative organ dysfunction, prolonged ICU stay and prolonged vasoactive support postoperatively. Finally, six out of eight patients who died were in the high emboli cohort group. Regarding the study’s evidence, future research is needed to confirm these results in prospective clinical trials on the relevance of routine use of TCD in cardiac surgery.

### Supplementary Information

**Additional file 1. Table S1.** Definition of Variables. **Table S2**. Oxygenator and CPB Weaning. **Table S3**. Intraoperative Hemodynamic Parameters Before CPB. **Table S4**. Characteristics of the Deceased Patients.

**Additional file 2. Fig. S1**. Weaning from CPB Protocol. **Fig. S2**. Transcranial Doppler Monitoring Devices and Signals. **Fig. S3**. Patient Screening. **Fig. S4**. HITS Distribution According to the Type of CPB Oxygenator. **Fig. S5**. HITS Distribution According to the Type of CPB Oxygenator and Stratify by HITS Category.

## Data Availability

Data and materials for the current study are available from the corresponding author on reasonable request.

## Abbreviations

2D: Two-dimensional
BMI: Body mass index
CABG: Coronary artery bypass graft
CBFV: Cerebral artery blood flow velocities
CI: Confidence interval
CPB: Cardiopulmonary bypass
CVP: Central venous pressure
EuroSCORE II: European System for Cardiac Operative Risk Evaluation II
HEM: High emboli material
HITS: High-intensity transient signal
ICU: Intensive care unit
IQR: Interquartile range
LEM: Low emboli material
LVEF: Left ventricular ejection fraction
MCA: Middle cerebral artery
MEM: Medium emboli material
NIRS: Near-infrared spectroscopy
NYHA: New York Heart Association
OR: Odds ratio
pEEG: Processed electroencephalography
Q: Quartiles
SD: Standard deviation
TCD: Transcranial Doppler
TPOD: Time of persistent organ dysfunction
