# Supplementary material for: Clinical relevance of transcranial Doppler in a cardiac surgery setting: embolic load predicts difficult separation from cardiopulmonary bypass
Source: J Cardiothorac Surg. 2024 Feb 13;19:90. doi: 10.1186/s13019-024-02591-4 (PMC10863099; doi:10.1186/s13019-024-02591-4)
Supplement: Supplementary file 1 — Additional file 1. Table S1. Definition of Variables. Table S2. Oxygenator and CPB Weaning. Table S3. Intraoperative Hemodynamic Parameters Before CPB. Table S4. Characteristics of the Deceased Patients. [file 13019_2024_2591_MOESM1_ESM.pdf]

ADDITIONAL FILE 1

Supplementary Table 1. Definition of Variables

Variable	Definitions
Body mass index ($kg \cdot m^{-2}$)	Weight /$(Height)^2$
Parsonnet	A score estimating the patient's risk of undertaking cardiac surgery and calculated by the clinician
EuroSCORE II	A score based on EuroSCORE II calculator model and calculated by the clinician before the surgery [1]
New York Heart Association III-IV	Patients classified with an NYHA of III or IV by the clinician
**Comorbidities**	
Pulmonary hypertension	Systolic pulmonary artery pressure of at least 30 mmHg or a mean pulmonary artery pressure >25mmHg, in the pre-operative evaluation. In this cohort, 253 patients had a pulmonary artery catheter
Acute kidney injury	Acute kidney injury is defined by Kidney Disease Improval Global Outcome (KDIGO) criteria [2]:<br>• Stage 1: ≥50% or 27 umol/L increases in serum creatinine<br>• Stage 2: ≥100% increase in serum creatinine<br>• Stage 3: ≥200% increase in serum creatinine or an increase to a level of ≥254 umol/L or dialysis initiation
Diabetes mellitus	Diabetes with drug or insulin requirement
Hypertension	History of treated or untreated hypertension
Heart failure with reduced ejection fraction	Left ventricular ejection fraction inferior to 50%

**Intraoperative data**	
Urgent surgery	Patients not electively admitted for operation but who require surgery on the current admission for medical reasons and cannot be discharged without a definitive procedure
Complex surgery	Combination of at least 2 procedures
High-intensity transient signal (HITS)	Both gaseous and solid emboli cause short intermittent amplifications in the ultrasound signal and are documented as a HITS
Intraoperative fluid balance (mL)	The total of volume administrated during the procedure (including crystalloids, colloids and blood transfusion products)
Intraoperative bleeding (mL)	Blood lost from wall suction bottles, weight of sponges, CPB related, and other sources
Cerebral Desaturation	A decrease of 20% from the value recorded at the beginning of the procedure or an absolute value of less than 50% at any time during the surgery.
**CPB separation** [3]	
Successful	The use of one or less pharmacological agent during CPB separation (less than one vasopressor or one inotropic agent)
Difficult	(1) The use of at least two different classes of vasoactive agents (inotropes or inhaled vasodilators and vasopressors) from CPB separation to the end of the surgery<br>(2) Return on CPB or the use of a mechanical circulatory support such as an intra-aortic balloon pump or a ventricular assist device for hemodynamic purposes

**Postoperative outcomes**	
Vasopressors	Norepinephrine, vasopressin, dopamine and phenylephrine were defined as vasopressors
Inotropes	Milrinone, dobutamine and epinephrine were considered as inotropic agents
Duration of mechanical ventilation (hours)	The time starting when the patient is intubated in the OR until the time the patient is extubated in the ICU
Delirium [4]	Defined as a diagnosis of acute confusion or encephalopathy at 24, 48- and 72-hours post-ICU arrival by the attending physician and/or a score of ≥4 on the Intensive Care Delirium Screening Checklist used by the nursing staff
Time of persistent organ dysfunction (TPOD) [5]	Time with Persistent Organ Dysfunction (POD) or death during the first 28 days (TPOD) is defined by Stoppe et al. as one or more of the following: mechanical ventilation; vasopressor therapy (ongoing need for vasopressor agents such as norepinephrine, epinephrine, vasopressin, dopamine >5 µg/kg/min, or phenylephrine >50 µg/min); mechanical circulatory support (ongoing need for mechanical devices such as extracorporeal membrane oxygenation (ECMO) or intra-aortic balloon pump; new continuous renal replacement therapy or new intermittent hemodialysis (first to last dialysis session). Therefore, TPOD represents the time for which the patient requires invasive life support after cardiac surgery. TPOD is a continuous variable representative of the burden of care and morbidity during the first 28 days following cardiac surgery and was chosen to circumvent issues arising for

	using other clinical endpoint such as ICU length of stay
Vasopressor time (hours)	The length of time the patient is on vasopressor therapy in the ICU. The time starting when the patient arrived in the ICU until patient discharge to the ward

Abbreviations: CPB: cardiopulmonary bypass; HITS: high-intensity transient signal; ICU: intensive care unit; OR: operating room

Supplementary Table 1 References

1. Nashef SA, Roques F, Sharples LD, et al. EuroSCORE II. Eur J Cardiothorac Surg. 2012;41(4):734-744.
2. Burton JO, Goldsmith DJ, Ruddock N, Shroff R, Wan M. Renal association commentary on the KDIGO (2017) clinical practice guideline update for the diagnosis, evaluation, prevention, and treatment of CKD-MBD. BMC Nephrology. 2018;19(1):240.
3. Denault AY, Tardif JC, Mazer CD, Lambert J. Difficult and Complex Separation from Cardiopulmonary Bypass in High-Risk Cardiac Surgical Patients: A Multicenter Study. J Cardiothorac Vasc Anesth. 2012;26(4):608-616.
4. Mailhot T, Cossette S, Lambert J, et al. Delirium After Cardiac Surgery and Cumulative Fluid Balance: A Case-Control Cohort Study. J Cardiothorac Vasc Anesth. 2019;33(1):93-101.
5. Stoppe C, McDonald B, Benstoem C, et al. Evaluation of Persistent Organ Dysfunction Plus Death As a Novel Composite Outcome in Cardiac Surgical Patients. J Cardiothorac Vasc Anesth.2016;30(1):30-38.

Supplementary Table 2. Oxygenator and CPB Weaning

	**Successful CPB weaning** (n=237)	**Difficult CPB weaning** (n=76)	***P value***
Fusion	97(40.9)	35(46.1)	0.864
FX15	42(17.7)	12(15.8)	
FX25	33(13.9)	14(18.4)	
INSPIRE 6F	13(5.5)	4(5.3)	
INSPIRE 8F	5(2.1)	1(1.3)	
RX15	20(8.4)	5(6.6)	
RX25	24(10.1)	4(5.3)	

Abbreviations: CPB: cardiopulmonary bypass; Fusion (Affinity Fusion, Medtronic, Minneapolis, USA); FX15 (CAPIOX FX15 Advance Oxygenator, Terumo, Ann Arbor (MI), USA); FX25 (CAPIOX FX25 Advance Oxygenator, Terumo, Ann Arbor (MI), USA); INSPIRE 6F (Inspire™, LivaNova, Mirandola (MO), Italy); INSPIRE 8F (Inspire™, LivaNova, Mirandola (MO), Italy); RX15 (CAPIOX® RX15, Terumo, Ann Arbor (MI), USA); RX25 (CAPIOX® RX25, Terumo, Ann Arbor (MI), USA)

Supplementary Table 3. Intraoperative Hemodynamic Parameters Before Cardiopulmonary Bypass

	**LEM (n=117)** (Below 133 HITS)	**MEM (n=119)** (> 133 and 413 HITS)	**HEM (n=118)** (> 413 HITS)	***P* value**
**Heart rate** (beats/min)				
Before CPB	58[49-65.5]	59[51-67]	60[53.75-70]	0.174
After CPB	73.5[66-80]	73[64.5-90.2]	75[64.5-82.25]	0.806
**Central venous pressure** (mmHg)				
Before CPB	11.06±4.09	12.11±4.44	11.87±4.45	0.153
After CPB	9.32±5.76	10.21±4.71	10.06±4.89	0.436
**Cardiac output** (L/min)				
Before CPB	3.60±1.03	3.86±1.11	4.08±1.29	0.013
After CPB	3.95±1.40	3.88±1.40	4.13±1.62	0.504
**Mean arterial pressure** (mmHg)				
Before CPB	76.57±9.88	77.40±12.89	75.26±11.12	0.352
After CPB	71.41±14.06	70.87±13.99	70.64±10.91	0.917
**Mean pulmonary arterial pressure** (mmHg)				
Before CPB	24.06±7.70	23.30±7.57	24.14±8.84	0.709
After CPB	21.49±7.64	21.30±7.55	22.81±6.61	0.309

Abbreviations: CPB: cardiopulmonary bypass; HEM: high quantity of embolic material; HITS: high-intensity transient signal; LEM: low quantity of embolic material; MEM: medium quantity of embolic material

Supplementary Table 4. Characteristics of the Deceased Patients

	**Non-deceased patients (n=346)**	**Deceased patients (n=8)**	***P value***
Age (years)	65.04±11.50	65.63±12.68	0.921
EuroSCORE II	1.50[0.9-3.1]	8.47[4.98-10.20]	<0.001
**Procedure**			
Urgent surgery [a]	60(17.3)	4(50)	0.038
Coronary artery bypass graft with CPB	137(39.6)	3(37.5)	0.605
Coronary artery bypass graft without CPB	40(11.6)	0	0.379
Simple valve	68(19.7)	1(12.5)	0.517
Complex surgery [a]	101(29.2)	4(50)	0.244
Maximum HITS	237.50[95-559.75]	725[298-4607.75]	0.013
Difficult weaning	69(22.6)	7(87.5)	<0.001
Cardiopulmonary bypass duration (min)	79[57-106]	125[102.5-232.25]	0.003
**Post-operative outomes**			
Duration of mechanical ventilation (hrs)	3[2-5]	1[0-8.25]	0.086
TPOD [a]	12.50	672[672-672]	<0.001
Acute kidney injury [a]	120(34.7)	6(75)	0.026
Vasopressor time (hrs)	10[2-30.50]	3.50[0-63.75]	0.389

[a] See Supplementary Table 1 for variable definition

Abbreviations: CPB: cardiopulmonary bypass; EuroSCORE: European System for Cardiac Operative Risk Evaluation; HITS: high-intensity transient signal; TPOD: time of persistent organ dysfunction
