# Supplementary material for: Clinical relevance of transcranial Doppler in a cardiac surgery setting: embolic load predicts difficult separation from cardiopulmonary bypass
Source: J Cardiothorac Surg. 2024 Feb 13;19:90. doi: 10.1186/s13019-024-02591-4 (PMC10863099; doi:10.1186/s13019-024-02591-4)
Supplement: Supplementary file 2 — Additional file 2. Fig. S1. Weaning from CPB Protocol. Fig. S2. Transcranial Doppler Monitoring Devices and Signals. Fig. S3. Patient Screening. Fig. S4. HITS Distribution According to the Type of CPB Oxygenator. Fig. S5. HITS Distribution According to the Type of CPB Oxygenator and Stratify by HITS Category. [file 13019_2024_2591_MOESM2_ESM.pdf]

ADDITIONAL FILE 2

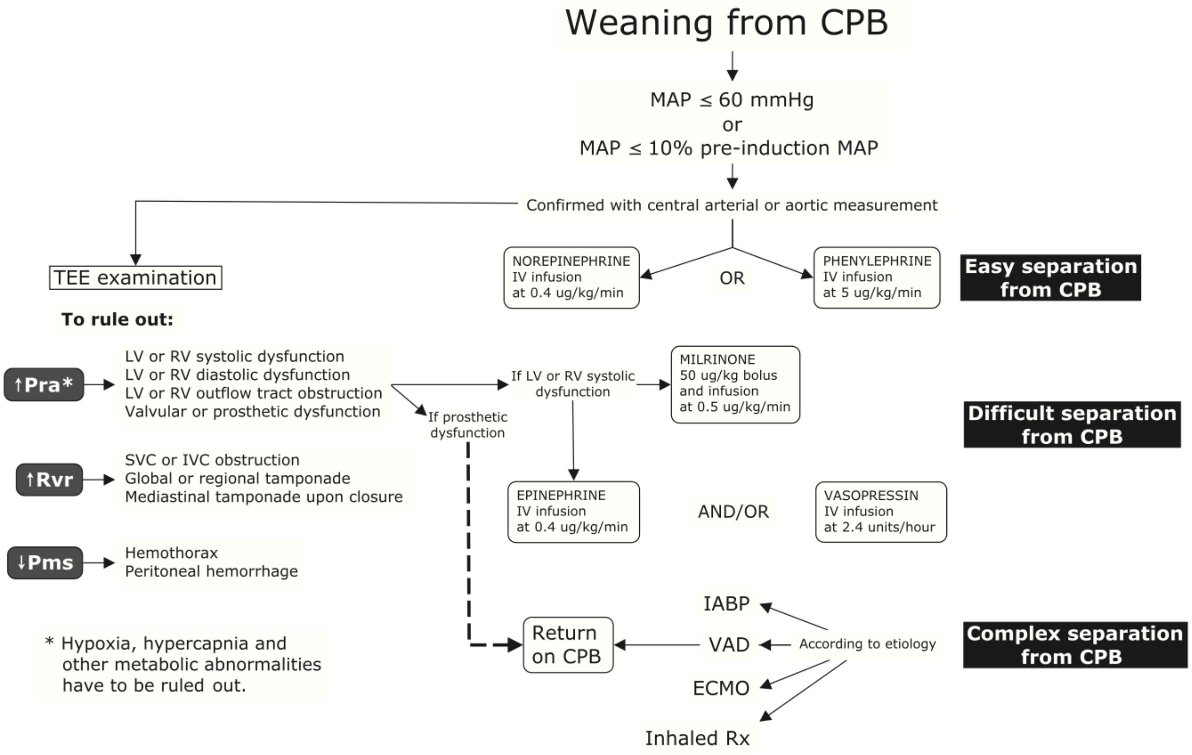

Supplementary Figure 1. Weaning from cardiopulmonary bypass (CPB) protocol. When the mean arterial pressure (MAP) is below 60 mmHg and confirmed with central measurement, transesophageal echocardiography (TEE) will be used to identify the etiology and rule out specific conditions associated with increased right atrial pressure (Pra), increased resistance to venous return (Rvr), and reduced mean systemic pressure (Pms). In the absence of a specific etiology, the first-line treatment (Rx) will include vasopressors (norepinephrine and phenylephrine). If this is insufficient and if there is evidence of left ventricular (LV) or right ventricular (RV) systolic dysfunction, inotropic agents (milrinone and/or epinephrine) will be used (difficult separation from CPB). If these are insufficient, other alternatives will be considered such as an intra-aortic balloon pump (IABP), LV or RV ventricular assist devices (VAD), extracorporeal membrane oxygenation (ECMO), and inhaled therapy (complex separation from CPB). IVC, inferior vena cava; SVC, superior vena cava. With the permission of Denault et al.[1]

Reference

1. Denault AY, Couture P, Beaulieu Y, Beaulieu Y, Haddad F, Deschamps A, et al. Right Ventricular Depression After Cardiopulmonary Bypass for Valvular Surgery. J Cardiothorac Vasc Anesth. 2015;29(4):836-844.

A B

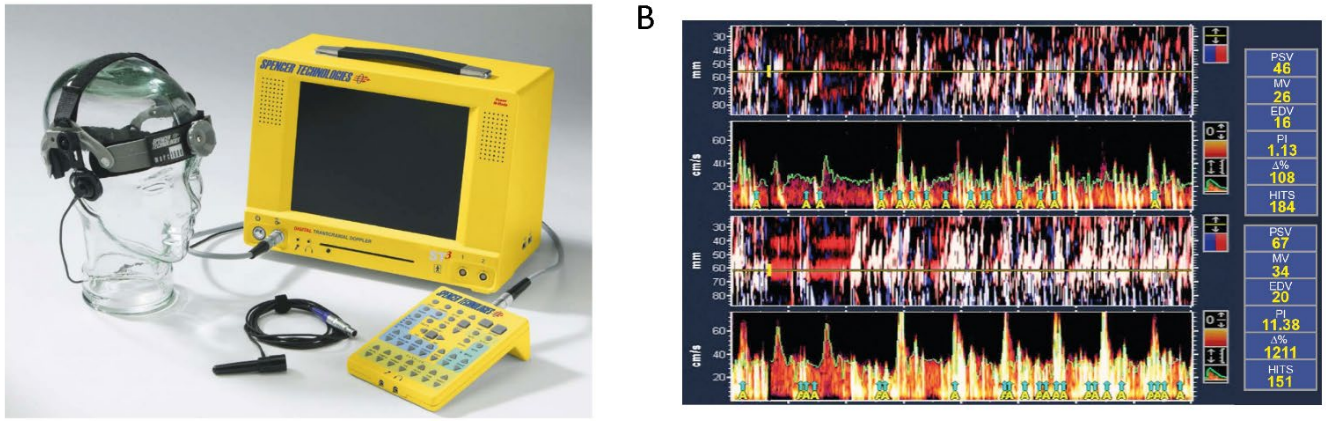

Supplementary Figure 2. (A) Transcranial Doppler monitoring devices (ST3, Spencer Technology, Seattle WA). (B) Transcranial Doppler signal in a 72-year-old man patient after aortic valve replacement and aortic surgery. Upon release of the aortic clamp, significant amount HITS were detected using transcranial Doppler. Note the appearance of HITS (yellow A and blue arrow).

Abbreviations: EDV: end-diastolic velocity; HITS: high-intensity transient signals; MV: mean velocity; PI: pulsatility index; PSV: peak systolic velocity; Δ%: relative change in mean peak systolic velocity over time. (Adapted with permission from Denault A, Vegas A, Lamarche Y, Tardif J, Couture P. *Basic Transesophageal and Critical Care Ultrasound*. New York, NY: Taylor and Francis, CRC Press, 2018).

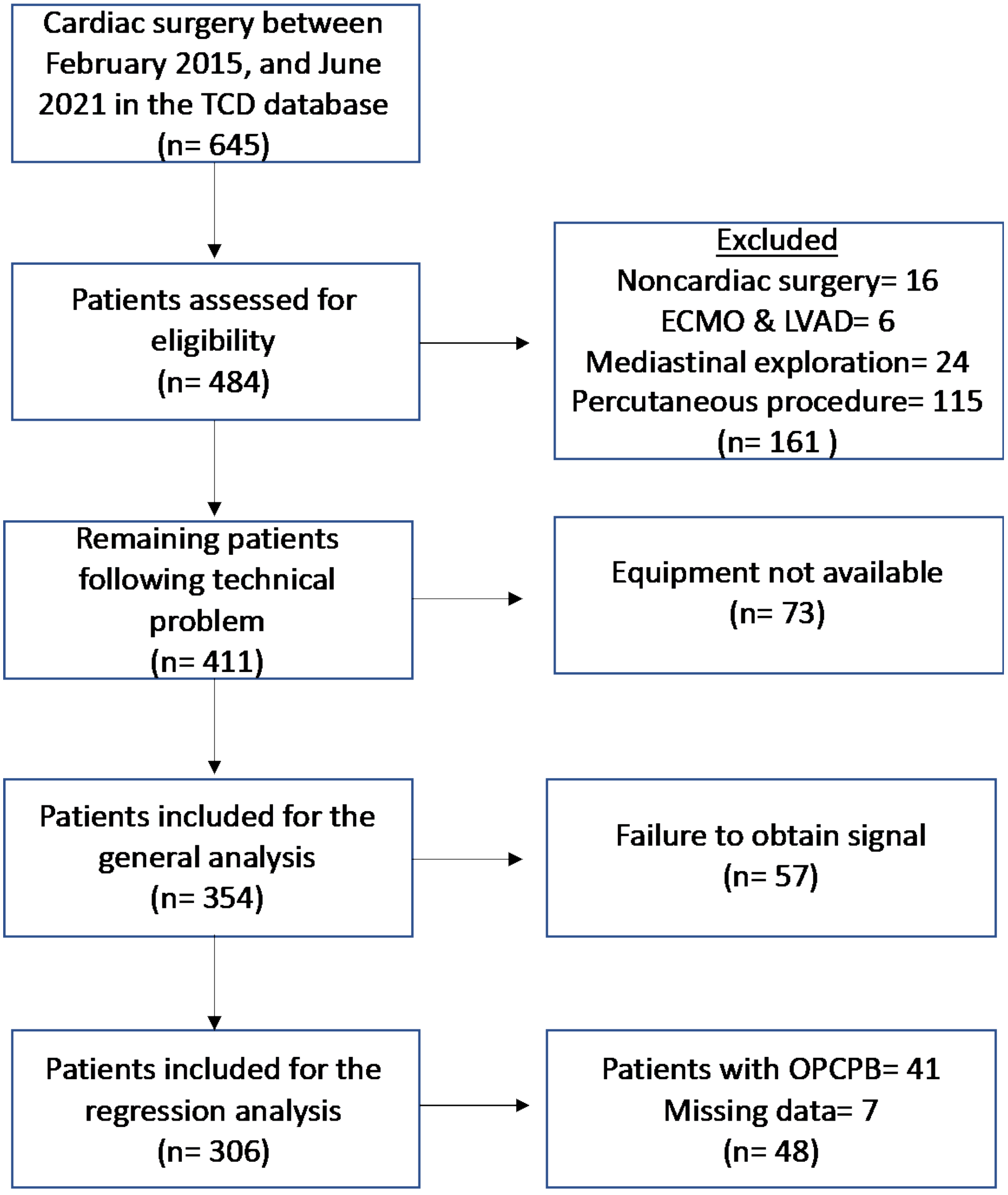

Supplementary Figure 3. Patient Screening.
Abbreviations: ECMO: extracorporeal membrane oxygenation; LVAD: left ventricular assist device; OPCPB: off pump cardiopulmonary bypass; TCD: transcranial Doppler.

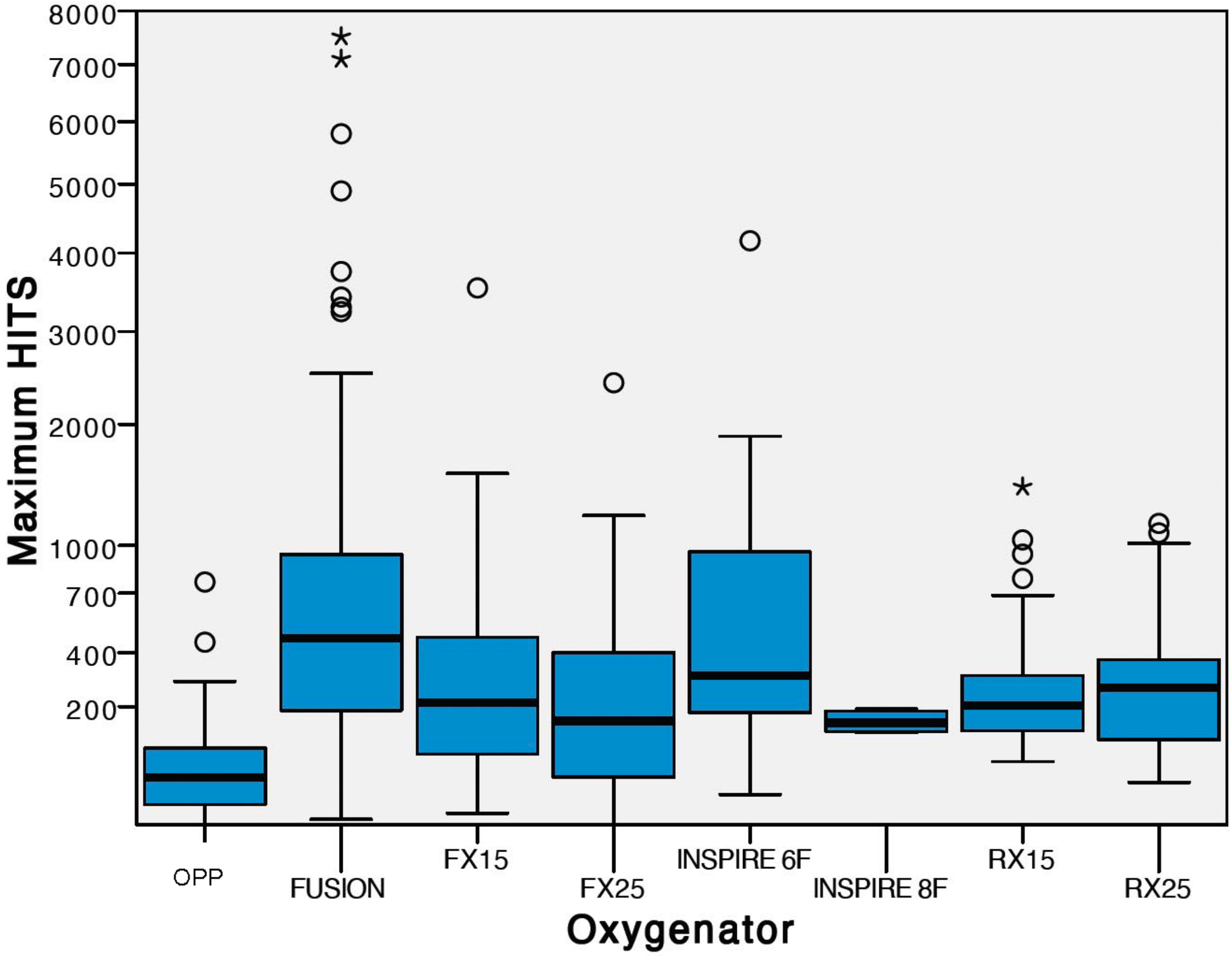

Supplementary Figure 4. HITS distribution according to the type of CPB oxygenator. The horizontal line is the median value, the box represents the inter-quartile range, and the bars represent the upper and lower 25th percentiles, excluding outliers.
* Outliers.
Abbreviations: CPB: cardiopulmonary bypass; OPP: off pump procedure; HITS: high-intensity transient signals; FUSION (Affinity Fusion, Medtronic, Minneapolis, USA);
FX15 (CAPIOX FX15 Advance Oxygenator, Terumo, Ann Arbor (MI), USA);
FX25 (CAPIOX FX25 Advance Oxygenator, Terumo, Ann Arbor (MI), USA);
INSPIRE 6F (Inspire^{TM}, LivaNova, Mirandola (MO), Italy);
INSPIRE 8F (Inspire^{TM}, LivaNova, Mirandola (MO), Italy);
RX15(Affinity arterial filter) (CAPIOX® RX15, Terumo, Ann Arbor (MI), USA);
RX25(Affinity arterial filter) (CAPIOX® RX25, Terumo, Ann Arbor (MI), USA).

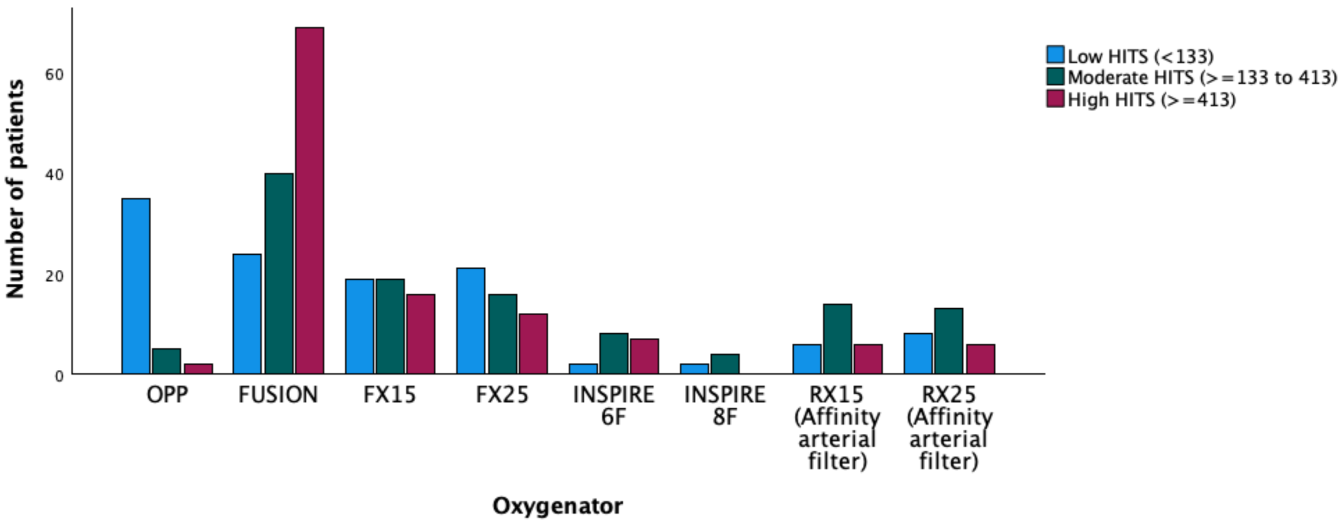

Supplementary Figure 5. HITS distribution according to the type of CPB oxygenator and stratify by HITS category.

Abbreviations: CPB: cardiopulmonary bypass; OPP: off pump procedure; HITS: high-intensity transient signals.
